# Editorial

**DOI:** 10.1038/sj.bjc.6602356

**Published:** 2005-01-14

**Authors:** A L Harris

It is a great honour for me to take over as Editor-in-Chief from Dr Robin Weiss.

Robin was the Director of the *Institute for Cancer Research* when I was a research fellow and gave me excellent advice on training in molecular biology. He has shown a clear vision in developing the *British Journal of Cancer* into multidisciplinary journal with a focus on research that aims to deliver benefits to cancer patients.

Under his leadership, the journal has become both more selective and more broadly based. A new section has been established specifically devoted to genetic approaches to the aetiology and progression of cancer and the new understanding that is developing from genomic studies. At the same time, these approaches have influenced all sections of the journal, which is now a natural home for well-planned rigorous studies, whether clinical, therapeutic or designed to illuminate basic processes in the disease, that are informed by the best of modern science. These are exciting times.

This explosion of ideas in cancer biology and therapies brings a pressing need for up-to-date overviews of new developments. Robin encouraged the commissioning of a very successful ongoing series of Minireviews in *BJC* designed to bring readers expert opinions and insights from leaders in the field.

He has encouraged debate within the pages of *BJC* and has led it through the earliest beginnings of web-based electronic publishing to a position where the rapid schedules of ‘advanced on-line publishing’ are bringing real benefits to the research community and offer great promise to those engaged in delivering the best care. I look forward to my own involvement in these developments.

Our understanding of the environmental and molecular causes of cancer, diagnostic imaging, pathology and therapeutic approaches to cancer have changed markedly in the last decade. These processes continue to develop at an accelerating rate, providing a challenge to all those involved in cancer research and therapy to keep up to date in their own and relevant fields, and to produce high-quality research.

The publication strategy of *BJC* reflects these changes, emphasising clinical translational research and preclinical investigations that are critical for new therapeutic approaches and drug development.

Clinical research covers a broad remit. Potentially the greatest gains in cancer survival, for the least cost, are informed by epidemiology and will involve changes in lifestyle. However, what determines individual responses to similar genetic or environmental changes? With the advent of high-throughput molecular analyses, as well as proteomics, the underlying mechanisms and pathways of relevance to the population and the individual can be investigated in a far more sophisticated way. Epidemiology has been an area strongly supported by the *BJC* and this will continue with alternate issues having an epidemiology section.

A major change in paradigm for cancer treatment has been to try and fit the therapy in the individual to match the oncogenic pathway in the cancer. Although recognised for nearly 30 years in management of breast cancer with use of the oestrogen receptor to select for hormonal therapy, it has only recently been applied in other situations as the therapeutic agents targeting specific pathways have become available. Herceptin and several antibody-targeted toxins and low molecular weight tyrosine kinase inhibitors have made a major impact on survival from cancer.

Thus, defining molecular pathways in cancer particularly in relationship to survival patterns of relapse and therapeutic response will be a mission for publications in *British Journal of Cancer*.

New diagnostic approaches including imaging, the relationship of imaging to tumour pathology and molecular mechanisms will be an important area for drug development and clinical practice. Gene array analyses and proteomics may provide the next generation of diagnostic classification, which will need to be related to therapeutic selection. Appropriate statistical analyses of survival, interactions of multiple pathways, sufficient patient numbers and duration of follow-up will be necessary to draw reliable conclusions. We will thus be including guidelines in these areas to help authors' submissions. Genetics is at the interfaces of cancer risk, diagnoses, outcome and therapeutic development and will continue to be an important area for the *BJC*.

The development of new drugs has become much more complex, highly regulated and expensive. With specific targets for new agents, demonstration that the target has been appropriately modified, for example, inhibition of receptor signalling either in the tumour or surrogate tissue is now an early decision point in drug development. The integration of pharmacokinetics with pharmacodynamic end points of the type described above is a key design for rational development of therapy. The *British Journal of Cancer* is keen to support publication of Phase I and II trials that incorporate novel features and end points in their designs, including imaging as well as surrogate end points or assays on tumour biopsies.

Critical preclinical experiments are, of course, essential to even reach the stage of Phase I trials, and there must be continued evaluation of mechanisms of action of existing agents, how they interact with each other and the new approaches. The *British Journal of Cancer* supports ethically conducted research in Experimental Therapeutics. More sophisticated models involving gene knockouts, mutations or genetically engineered lines that provide new models to assess therapeutics or mechanisms of disease will be supported and provide an opportunity for validating clinical end points. This section will be renamed Translational Therapeutics to emphasise the translational nature of the work.

Final adoption of a new therapy and the optimisation of existing therapy depend on the outcome of randomised Phase III trials. Methodology for their analysis continues to undergo development and analysis of molecular pathways in the patient and tumour will almost certainly contribute to understanding of who benefits most from treatment. Ultimately, selection as is currently applied to antibody-targeted therapy will be necessary to use combination single agents appropriately in adjuvant therapy. Thus, the *BJC* supports Phase III trials, particularly if factors relating to outcome can be defined.

We must not forget the impact of these treatments on patients’ quality of life and their agenda. The *BJC* continues to invite papers in this area.

Journals have become more specialised, and there are specific journals for every pathway or clinical situation described above. It thus becomes increasingly important to have a journal that can cover a breadth of cancer research relevant to translational scientists and clinicians, in order to foster communication, the generation of crossdisciplinary knowledge and ideas, to enable investigators to keep up to date in areas in addition to their specific focus. We therefore think the Minireviews designed to be short, pithy and written by the scientists who did the research will continue to be an important regular feature. They will address basic molecular biology of translational relevance and the science behind new therapeutic developments.

I hope therefore that the *BJC* will continue to flourish as a cross and multidisciplinary journal, and that it will become the journal of choice for translational cancer research.

